# Measuring the importance of influencing factor for COVID-19 vaccination intention in China

**DOI:** 10.3389/fpubh.2023.1191401

**Published:** 2023-06-27

**Authors:** Yue Su, Sijia Li, Jia Xue, Ang Li, Tingshao Zhu

**Affiliations:** ^1^CAS Key Laboratory of Behavioral Science, Institute of Psychology, Chinese Academy of Sciences, Beijing, China; ^2^Department of Psychology, University of Chinese Academy of Sciences, Beijing, China; ^3^Department of Social Work and Social Administration, The University of Hong Kong, Pokfulam, Hong Kong SAR, China; ^4^Factor-Inwentash Faculty of Social Work, University of Toronto, Toronto, ON, Canada; ^5^Faculty of Information, University of Toronto, Toronto, ON, Canada; ^6^Department of Psychology, Beijing Forestry University, Beijing, China

**Keywords:** COVID-19 vaccine, vaccine intention, vaccine willingness, influencing factor, scale development, vaccine hesitancy, factor importance

## Abstract

**Background:**

Vaccination is considered an effective approach to deter the spread of coronavirus disease (COVID-19). However, vaccine hesitancy is a common issue that makes immunization programs more challenging. To promote vaccination in a targeted and efficient way, this study aims to develop and validate a measurement tool for evaluating the importance of influencing factors related to COVID-19 vaccination intention in China, and to examine the demographic differences.

**Methods:**

In study 1, we developed a Factor Importance Evaluation Questionnaire (FIEQ) based on semi-structured interview results and used exploratory factor analysis (EFA) to explore its factor structure. In study 2, we verified the four-factor structure of FIEQ by confirmatory factor analysis (CFA). We then administered FIEQ to Chinese participants and conducted a student t-test and analysis of variance to examine the differences in the importance evaluation of factors based on gender and educational level.

**Results:**

In study 1, we developed a four-factor construct and retained 20 items after EFA (*N* = 577), with acceptable reliability (alpha = 0.87) and validity. In study 2, we found that the model fit was good (*χ*2 = 748.03 (162), *p* < 0.001, GFI = 0.949, RMSEA = 0.049, SRMR = 0.048, AGFI = 0.934), and reliability was acceptable (alpha = 0.730) (*N* = 1,496). No gender difference was found in factor importance. However, individuals with different educational levels reported significantly different importance evaluations of three factors, including perceived benefits and social norms (*F* = 3.786, *p* = 0.005), perceived influences from reference groups (*F* = 17.449, *p* < 0.001), and perceived risks (*F* = 2.508, *p* = 0.04).

**Conclusion:**

This study developed and validated FIEQ for measuring the importance of influencing factors related to the COVID-19 vaccination intention in Chinese participants. Moreover, our findings suggest that the educational level may play a role in how individuals evaluate the importance of factors. This study provides insights into the concerns that individuals have regarding vaccination and offers potentially effective and targeted strategies for promoting COVID-19 vaccination.

## 1. Introduction

The COVID-19 pandemic has been a global concern for over 3 years, resulting in various challenges such as travel restrictions, loss of life, and economic stagnation. Therefore, it is vital to promote efficacious preventive measures against COVID-19 to control and prevent the spread of the pandemic. One of the key approaches is the vaccination campaign (1). However, vaccine hesitancy is a common problem that makes immunization programs more challenging in many countries (2).

Vaccine hesitancy is defined as the “delay in acceptance or refusal of vaccines despite availability of vaccine services” (3). Some sociodemographic characteristics are related to COVID-19 vaccine hesitancy, such as gender (4), age (5), educational level (6), and private health insurance (7). Besides, some factors pertaining to individuals’ confidence and beliefs regarding the COVID-19 pandemic and the vaccine also have impacts on vaccination intentions, including perceived benefits and barriers (8), and contribution to disease control (9).

These influencing factors may work as health communication guidance to increase vaccine uptake. However, there remains uncertainty surrounding the effectiveness of these efforts as it is unclear to what extent these factors affect the participants. For example, vaccine safety may not be equally prioritized by all individuals. One study designed messages to address the concerns about vaccines and failed to find any effects of messages on vaccination intentions (10). The possibility of including participants who did not have concerns about the development or safety of COVID-19 vaccines may confound the results, thereby leading to failure of communication (10). Messages designed to address the concerns about vaccines may be more powerful if targeted to individuals with significant concerns. Another study examined the effects of reference groups and shared similar findings as the reference group may not be an important factor for all participants (11). Furthermore, the different groups would exhibit unique patterns of influencing factors and these factors were sensitive to contextual differences, such as key workers and non-key workers in UK (12). Thus, public health messaging needs to address the most pertinent concerns of individuals to ensure effective vaccination campaigns (12).

Therefore, we need to figure out the factors that are important to individuals, as this would provide a detailed understanding of vaccination intentions and help tailor vaccine promotion messages. However, little is known about how these factors would be weighed by individuals. Though previous researchers have attempted to develop scales to assess the importance of COVID-19 vaccine-related factors, they have failed to validate the psychometric properties (13). To the best of our knowledge, no validated measurement tool has been used in previous studies. Hence, we aim to develop the Factor Importance Evaluation Questionnaire (FIEQ) for the COVID-19 vaccine, a psychometrically sound questionnaire that can comprehensively investigate the factor importance related to the COVID-19 vaccine and identify the patterns of important factors for individuals.

How COVID-19 vaccine-related influencing factors are weighed seems to be associated with personal variables, such as demographic characteristics. As demographic characteristics are commonly used to explain variation in COVID-19 vaccination beliefs, it is plausible that these characteristics may also influence the factor importance evaluation. For instance, gender and educational level are important attributes related to COVID-19 vaccine hesitancy (6). Male gender and low education level have been found to be relevant factors that may influence COVID-19 vaccine hesitancy in China (14, 15). These findings indicated that perceptions of the COVID-19 vaccine may differ across genders and educational levels, leading to varying motivators for vaccination uptake. However, few studies have explored whether these demographic characteristics could influence the importance evaluation of influencing factors. Therefore, we aim to confirm this speculative relationship through difference tests. This knowledge has important implications for vaccine promotion as it could help to target specific demographic groups and obtain detailed segments.

The purpose of our study is to focus on the salient concerns and refine possible directions for policy development by investigating COVID-19 vaccine-related factor importance for individuals. Following the standard process of scale development, this study aims to build a good construct of influencing factors of COVID-19 vaccination and develop a psychometrically sound questionnaire - Factor Importance Evaluation Questionnaire (FIEQ) for COVID-19 vaccine -for evaluating the importance of influencing factors pertinent to COVID-19 vaccination intention. We further investigate the differences based on gender and education level when individuals weigh influencing factors.

## 2. Study 1

### 2.1. Methods

To develop and evaluate FIEQ, this study was conducted in three phases: questionnaire development, participant recruitment, and data analysis.

#### 2.1.1. Questionnaire development

This study developed FIEQ based on the research of Su and colleagues (16), who conducted semi-structured interviews to identify the key points that would increase their COVID-19 vaccination intentions. Thematic analysis of the interviews resulted in 31 codes, such as vaccine safety, vaccine effectiveness, and risk perception, that participants deemed important to increase vaccination willingness.

In this study, we drafted the original version of FIEQ based on these 31 codes. Specifically, we developed one question from each code obtained in the interviews, resulting in a 31-item questionnaire. To ensure content validity, we invited 13 experts from the Department of Psychology, including 12 graduate students and one teacher, to form an evaluation committee to review the questions for grammar, readability, and accuracy. They rated the degree of relevance of each item and provided their suggestions and comments. We followed the popular method recommended by Davis to calculate content validity (17). Specifically, the content validity index (CVI) can be calculated as evidence of content validity (18). S-CVI (the scale-level content validity index) is 0.955, which met a satisfactory level. Besides, nearly all items scored highly on CVI, with scores ranging from 0.846 to 1. Only one item (Item 26) got a score of 0.692, which was still relatively close to the acceptance threshold of 0.7. Therefore, we decided to keep all items and make some modifications to enhance statement quality. Two researchers (including the first author) gathered all suggestions and reached an agreement on rephrasing and finalizing the questionnaire items. Following the above steps, the first draft of FIEQ was obtained (Please see Appendix 1 for the first draft of FIEQ in both Chinese and English translations).

#### 2.1.2. Recruitment

An online questionnaire was administered to collect survey data for this cross-sectional study in September 2021, coinciding with the third wave of the pandemic (19). The first draft version of FIEQ was published on the iSurveylink platform.^1^ People responded to the survey voluntarily. ISurveylink is a widely-used online research service provider in China (20–22), with a sample data pool of over 6.59 million users. Participants who met the age requirement of 18 years and above and were Chinese residents were eligible for participation, while those who were unable to understand written Chinese were excluded.

We used the seven-point Likert-type scale in the first draft of FIEQ, under which one meant strongly disagree, four meant not sure, and seven meant strongly agree. Participants were required to rate their degree of agreement to all questions on a scale from one to seven.

#### 2.1.3. Statistical analysis

We conducted descriptive analyses using Statistical Product Service Solutions (SPSS). The Kaiser–Meyer–Olkin (KMO) test was used to examine the sampling adequacy, while Bartlett’s test of sphericity was used to check the sufficiency of inter-item correlations (23). We then conducted the exploratory factor analysis (EFA) using SPSS. Principal component analysis was employed to extract factors, while the Caesar normalization maximum variance was used as the rotation method to improve the interpretability of the solution (24). Based on the EFA results, we removed 11 items. Then, we calculated Cronbach’s alpha score and McDonald’s omega score to measure the reliability of the new version of FIEQ without these 11 questions.

#### 2.1.4. Ethical considerations

Our study was approved in advance by the Ethics Committee of the Institute of Psychology, Chinese Academy of Sciences with the approved number H15009. All participants provided their informed consent, and the data collected in this study was anonymous.

### 2.2. Results

We obtained a total of 667 responses *via* the iSurveylink platform. To ensure the quality of the collected data, we undertook a filtering process and excluded questionnaires displaying abnormal answer times. Specifically, answer times exceeding 60 min or falling below 3 min were deemed abnormal and removed from the dataset, leaving us with a final sample of 577 participants. The demographic information of our data sample can be found in Table 1. For descriptive information of our survey results, please refer to Appendix 2.

**Table 1:** The demographic information of our sample.

Characteristic	Mean (SD) / *n* (%)
Age	40.7(15.1)
Gender	
Male	273(47.3%)
Female	304(52.7%)
Education	
Junior high school and below	23(4.0%)
High school (including technical secondary school)	105(18.2%)
College (three-year or two-year college diploma)	155(26.9%)
Bachelors	196(34.0%)
Masters and above	98(17.0%)

#### 2.2.1. Exploratory factor analysis

Results showed that the KMO value was 0.93 and Bartlett’s test of sphericity was significant (*χ*2 = 6382.59; *p* < 0.001), which indicated factor analysis was appropriate for our sample data (25). EFA was performed and resulted in five factors (eigen values >1). We considered factor retention based on the number of items per factor since it is a conventional criterion (26, 27). It was recommended to remove factors with fewer than three items (28–30). Results indicated that Factor 5 had only two items: Item 3 and Item 21. We then examined the exact meanings of these two items and found they shared similarities in language instead of contents, which indicated that Factor 5 cannot be accepted as a meaningful factor. Taking these two points into consideration, we removed Factor 5 from our study. For the stability of the factor solution, it is typically recommended to delete items with low factor loading (31, 32). Consequently, eight items (Item 9, 10, 11, 13, 17, 22, 26, 28) were excluded due to their factor loading being less than 0.50. Item 5 was also removed since it exhibited cross-loading on two factors and we aimed to ensure a clear factor structure. Specifically, their loadings on Factor 2 and Factor 4 were quite similar and the difference between these loadings was fewer than 0.2. Then, we examined the scree plot to ensure the appropriateness of a four-factor solution.

The final version of FIEQ contained 20 items with four factors, which explained approximately 46.0% of the total variation. The factors were as follows: Factor 1 included seven items with the theme “Perceived benefits and social norms.” Factor 2 comprised five items with the theme “Perceived influences from reference groups.” Factor 3 involved five items with the theme “Perceived risks,” whereas Factor 4 consisted of three items with the theme “Vaccine safety.” The factor loadings for EFA were presented in Table 2, which demonstrated that all the items in FIEQ had factor loadings above 0.52.

**Table 2:** Summary of EFA results.

Item ^a^	Factor 1	Factor 2	Factor 3	Factor 4
Item 23	0.72			
Item 24	0.67			
Item 29	0.67			
Item 16	0.62			
Item 31	0.60			
Item 8	0.55			
Item 25	0.55			
Item 19		0.69		
Item 18		0.68		
Item 7		0.66		
Item 27		0.64		
Item 15		0.60		
Item 30			0.65	
Item 2			0.62	
Item 20			0.55	
Item 12			0.53	
Item 14			0.52	
Item 6				0.69
Item 4				0.65
Item 1				0.56
Cronbach alpha	0.827	0.769	0.730	0.631
McDonald’s omega	0.833	0.778	0.731	0.637

Extraction method: principal component analysis method.

Rotation method: Caesar normalization maximum variance method.^b^

^a^ Only factor loadings above 0.50 in each factor are presented.

^b^ The rotation has converged after 25 iterations.

#### 2.2.2. Reliability

We measured reliability by calculating Cronbach’s coefficient alpha and McDonald’s omega. The alpha score of FIEQ was 0.873, which was of high reliability and accepted in psychological measurement. McDonald’s omega was 0.878, indicating good internal reliability. The Cronbach alpha score and McDonald’s omega score for each factor were also shown in Table 2, which indicated acceptable reliability for every factor.

#### 2.2.3. Validity

We examined the discriminant validity by comparing the square root of average variance extracted (AVE) and correlation coefficients. Table 3 presented the root value of each factor’s AVE and the correlation coefficient between factors. Overall, the root values of AVE scores of all factors were larger than their correlation coefficients, which indicated a good discriminant validity for FIEQ.

**Table 3:** The results of AVE and correlation coefficient.

	Factor 1	Factor 2	Factor 3	Factor 4
Factor 1	0.630			
Factor 2	0.524^**^	0.656		
Factor 3	0.571^**^	0.327^**^	0.577	
Factor 4	0.366^**^	0.215^**^	0.536^**^	0.635

** Correlation is significant at the 0.01 level (2-tailed).

The number on the diagonal line is the root value of the factor’s AVE.

## 3. Study 2

### 3.1. Methods

In study 2, we aimed to validate FIEQ and explore differences in factor importance based on a new data sample, which contained more participants from a diverse population. This cross-sectional study collected survey data through an online questionnaire. Considering that gender and educational level are commonly acknowledged factors associated with vaccination intention (33), identifying the appropriate target group based on these attributes can contribute to a more effective vaccination promotion.

#### 3.1.1. Recruitment

We recruited participants by releasing questionnaires on the iSurveylink in October and November 2021, coinciding with the third wave of the pandemic (19). FIEQ consisted of 20 items, which were retained by EFA in Study 1. We recoded these items from 1 to 20 (please see Appendix 3) and used the seven-point Likert-type scale which was the same as Study 1. Each participant was required to respond to every item in FIEQ and rate their degree of agreement from one (strongly disagree) to seven (strongly agree). Our study was approved in advance by the Ethics Committee of the Institute of Psychology, Chinese Academy of Sciences (approved number: H15009).

#### 3.1.2. Statistical analysis

We used SPSS to conduct descriptive analyses and compute Cronbach’s alpha score and McDonald’s omega score. Furthermore, we performed confirmatory factor analysis (CFA) using the Statistical Product and Service Software Automatically (SPSSAU). SPSSAU is a web-based data science algorithm platform tool that can be used to conduct multiple data analyses, such as Analysis of variance (ANOVA) and regression analysis. We chose to employ maximum likelihood estimation in CFA. This estimation strategy performed well when the model was reasonably accurate and the sample size was reasonably large (34), which was widely used in CFA studies (35–37). We adopted the acknowledged criteria to assess the model fit (38, 39). Specifically, an acceptable model fit was suggested if the CMIN/DF value was less than five, the values of the goodness of fit index (GFI) and adjusted goodness of fit index (AGFI) were higher than 0.90, the value of root mean square error of approximation (RMSEA) was below 0.10 and the standardized root mean square residual (SRMR) value was below 0.05.

Researchers have previously recommended treating Likert scale responses as continuous variables and calculating a total score or mean score for each factor (40). Therefore, we calculated the mean score of item responses for each factor, an operation commonly performed in previous studies containing multiple domains (41–43). Subsequently, a student’s t-test was conducted to explore the differences in the evaluation of factor importance between males and females, while ANOVA with *post hoc* Bonferroni was employed to investigate differences among people with different education levels.

### 3.2. Results

A total of 1,589 participants took part in this study, with 93 questionnaires being excluded due to their abnormal answer time. The final sample size consisted of 1,496 participants, and a descriptive analysis of the sample was shown in Table 4. For detailed information of the FIEQ responses, please refer to Appendix 4.

**Table 4:** Descriptive analysis of the data sample in study 2.

Characteristic	Mean (SD) /*n* (%)
Age	42.0(14.6)
Gender	
Male	747(49.9%)
Female	749(50.1%)
Education	
Junior high school and below	149(10.0%)
High school (including technical secondary school)	277(18.5%)
College (three-year or two-year college diploma)	431(28.8%)
Bachelors	571(38.2%)
Masters and above	68(4.5%)
Factors	
Factor 1	6.0(0.8)
Factor 2	5.4(1.0)
Factor 3	6.2(0.6)
Factor 4	6.1(0.7)

The four-factor structure of FIEQ was verified *via* CFA after adding two covariance errors and fit indicators were shown in Table 5. Besides, we used Cronbach’s alpha and McDonald’s omega to measure internal consistency. The Cronbach alpha score was 0.730, which was acceptable reliability in the psychometric field. McDonald’s omega was 0.703, indicating adequate internal consistency (44).

**Table 5:** Fit indicators for FIEQ.

Model	*χ*2	df	GFI	RMSEA	SRMR	AGFI
Four-factor model	748.03	162	0.949	0.049	0.048	0.934

For the difference examination of factor importance in terms of gender, results were shown in Table 6. No significant difference was found in each factor between males and females.

**Table 6:** *T*-test result.

	Male(*n* = 747), mean (SD)	Female(*n* = 749), mean (SD)	*t*	*p* value
Factor1	5.9(0.6)	5.9(0.7)	1.013	0.31
Factor2	5.0(0.9)	5.1(0.9)	−1.603	0.11
Factor3	6.2(0.6)	6.2(0.6)	−0.065	0.95
Factor4	6.0(0.7)	6.1(0.8)	−1.225	0.22

Table 7 showed the differences in factor importance among individuals with different levels of education. Three factors showed significant differences across various education levels, including Factor 1 (*F* = 3.786, *p* = 0.005), Factor 2 (*F* = 17.449, *p <* 0.001), and Factor 3 (*F* = 2.508, *p* = 0.04). Specifically, individuals with bachelor’s degrees reported significantly higher scores on both Factor 1 and Factor 3 compared to those with a high school diploma. Regarding Factor 2, people with bachelor’s degrees, and master’s degrees and above scored higher than those with a high school level of education or below. Additionally, people with bachelor’s degrees reported Factor 2 as more important than individuals with a college degree. And individuals with a college degree scored higher on Factor 2 than those with junior high school education and below.

**Table 7:** ANOVA result.

	mean (SD)					*F*	*p* value
	Junior high school and below (*n* = 149)	High school (*n* = 277)	College (*n* = 431)	Bachelors (*n* = 571)	Masters and above (*n* = 68)		
Factor1	5.87(0.65)	5.81(0.65)	5.93(0.70)	5.99(0.59)	5.88(0.63)	3.786	0.005
Factor2	4.71(1.10)	4.88(0.91)	5.04(0.89)	5.26(0.75)	5.24(0.81)	17.449	<0.001
Factor3	6.21(0.60)	6.15(0.66)	6.25(0.59)	6.28(0.58)	6.27(0.62)	2.508	0.04
Factor4	5.96(0.74)	6.03(0.76)	6.03(0.72)	6.07(0.75)	6.15(0.68)	0.919	0.45

## 4. Discussion

This study explored how influencing factors related to COVID-19 vaccination intention were weighed among Chinese participants during the COVID-19 pandemic. Specifically, a measurement tool named FIEQ was developed and validated to measure the importance of COVID-19 vaccine-related factors. By conducting a factor analysis, the factors people consider when deciding to get vaccinated were grouped into four main categories. No significant difference in factor importance was found between male and female participants. However, people with different education levels showed varied evaluations of factor importance.

***Perceived benefits and social norms.*** The first factor, “perceived benefits and social norms,” consists of seven items. This factor encompasses the benefits people expect to receive after vaccination, such as financial incentives, special badges, and travel convenience, as well as certain rules or behaviors that are deemed required and encouraged within a community or society. Examples of this factor include statements like “If my Health Code will become different after being vaccinated with the COVID-19 vaccine, then I will be more willing to get the vaccine” and “The government and official media’s policy of advocating vaccinations against COVID-19 will increase my willingness to vaccinate.” Researchers have acknowledged the rationality of providing financial incentives as it could compensate for the indirect expenses of getting vaccinated and motivate some individuals to overcome their inertia (45). Moreover, social norms have been identified as a crucial factor that affects people’s vaccination intention in previous research (46, 47). These social norms may stem from recommendations from the government, family, or friends (46, 47), which aligns with the findings of our study.

***Perceived influences from reference groups.*** The second factor, “perceived influences from reference groups,” includes five items. This factor underscores the influence of individuals and groups that people value and endorse. When individuals consider getting vaccinated, they value and seek the opinions of these people or groups. Reference groups are groups that indirectly or directly affect a person’s values, attitudes, and behaviors, including friends, teachers, or public figures. For example, “Compared to the official media, I prefer to believe in the opinions of some self-media figures, who have a certain number of fans and are engaged in science-related content, or netizens who have been vaccinated against COVID-19.” Other studies have yielded comparable findings, such as mistrust in authority (48) and reluctance to believe traditional information sources (49). These results emphasize the importance of unofficial media and manifest the low credibility levels of some groups, which is consistent with our results. Furthermore, several studies have indicated that the opinions of the majority hold significant sway (9, 50), findings echoed in our own research.

***Perceived risks.*** The third factor, named “perceived risks,” is composed of five items. This factor pertains to the perceived risks associated with COVID-19 and the vaccination. Specifically, the perceived risk of COVID-19 vaccination itself is a significant influencing factor, and the assessment of risk by one’s surroundings could also impact the attitude regarding vaccination to some extent. For example, “If the domestic epidemic outbreaks again and the risk of infection increases, then my willingness to vaccinate against COVID-19 will be greatly improved.” Recent studies have yielded similar findings (51, 52). Moreover, Wu and colleagues have proposed that weighing the possibility of vaccine side effects against one’s perceived risk of contracting disease is a key decision-making process when considering vaccination (53). This opinion underscores the cognitive process of assessing perceived risks associated with vaccination.

***Vaccine safety.*** The fourth factor, labeled “vaccine safety,” contains three items. This factor stresses the significance of how individuals perceive the safety of COVID-19 vaccines when deciding to receive them. Contraindications, explicit age-group limitations, and potential side effects can all influence an individual’s judgment regarding vaccine safety. For example, “I hope that specific explanations to certain people who have vaccination restrictions could be given when promoting the COVID-19 vaccine, such as the reasons why older adults were not allowed to vaccinate before.” Vaccine safety has also been recognized as a key influencing factor in vaccination willingness in numerous previous studies (51, 54, 55). As such, researchers have emphasized the need to enhance the perception of vaccine safety to promote vaccine uptake (56). To accurately reflect the safety profile of the vaccine, researchers suggest implementing surveillance programs of adverse events (57, 58) and systematic use of causality assessment (59). By providing scientific and practical evidence for limitations and possible side effects, these programs could bolster the perceived vaccine safety and increase public confidence in the vaccination program.

In this study, we found no significant differences between males and females in terms of evaluating factor importance. This finding indicated that gender might not influence how people weigh factors related to COVID-19 vaccination intention. It implies that attention should be given to other characteristics, such as the educational level, when designing tailored public health communication messages. Besides, *perceived benefits and social norms, perceived influences from reference groups,* and *perceived risks* were found to be more highly valued by people with higher educational levels. Education offers various health resources that may be contributing to people’s responses to COVID-19 vaccine-related influencing factors (60). As researchers have suggested, education is related to knowledge, credentials, social networks, cognitive resources, and cultural resources (60, 61), which provide a plausible explanation for the differences in factor importance. However, people with different education levels did not evaluate the importance of *vaccine safety* differently. This finding suggested that vaccine safety seemed to be a common concern regardless of educational level. Given that the COVID-19 vaccine has been developed in a considerably short time frame, and its long-term effects are still unknown, such concern may be understandable.

A previous study examined the effects of different message appeals for COVID-19 vaccine uptake and suggested that preferences for particular appeals may vary by different audience segments (62). These findings inform the heterogeneity of factor importance among subgroups and complement the rationality of questionnaire development. To the best of our knowledge, the FIEQ developed in our study is the first validated measurement tool to measure how individuals weigh influencing factors associated with COVID-19 vaccination intention. This study provides a comprehensive construct containing four key factors associated with COVID-19 vaccination intention. FIEQ could be used to examine the effects of each key factor by self-report.

This study contributes to a more targeted approach to promoting vaccine uptake. First, although many influencing factors have been identified in previous studies, determining the relative importance of each factor for individuals is still difficult. In the absence of such information, practitioners would be uncertain about where to direct their resources and what should be prioritized (63). We suggest the FIEQ developed in our study could provide evidence-based instruction for selecting effective influencing factors and help address the concerns in an individualized way. For example, public health workers can use FIEQ to identify essential factors for a specific group. Supposing that people score higher on “perceived risks,” public health workers could prioritize explaining and clarifying the risks of the COVID-19 vaccine in the subsequent vaccination campaign. Besides, findings in this study also indicate that three key factors would work more effectively for people with higher education levels than those with lower education levels.

Furthermore, considering the importance level could help clarify the effects of influencing factors in communication intervention studies. When researchers plan to examine whether messages containing an influencing factor would be helpful in increasing vaccination intention in an experiment, we suggest researchers measure the importance of the influencing factor to confirm the homogeneity among participants, which might enhance the impact of the factor. To conclude, this study could contribute to a more precise and nuanced understanding of people’s perspectives on vaccine uptake and provide a further impetus to targeted vaccination interventions.

Our study also has some limitations. First, FIEQ is developed for the COVID-19 vaccine and may not be generalizable to other infectious diseases, but our method could serve as a useful point of reference for future research on other diseases. Second, as our study was conducted in China, applying FIEQ elsewhere requires prudence. Nevertheless, with proper modification and cross-cultural adaptation, FIEQ may still convey meaningful insights into the critical motivators of COVID-19 vaccination. Besides, the sample used in this study was limited to people with access to the Internet and electronic devices. Therefore, it may introduce a bias related to socioeconomic status and education and may not be fully representative of the Chinese population, thus, restricting the generalizability of our findings. Future studies should test the psychometric properties of FIEQ with more diverse samples. Furthermore, researchers should consider more personal characteristics, such as income, residential location, and occupation, to better clarify the effect of each variable and control the potential confounding factors. Additionally, vaccination behavior may be an important variable for assessing influencing factors and should be accounted for in future studies. While our study primarily focused on identifying communication contents for vaccination, we acknowledge that the means of communication matters as well. In this regard, future researchers should consider the effects of mass media on vaccination promotion.

## 5. Conclusion

In this study, we explored how the importance of influencing factors related to COVID-19 vaccination intention would be weighed and examined the differences in gender and educational level among Chinese participants. First, we developed the Factor Importance Evaluation Questionnaire, a validated measurement tool with a four-factor construct. Then, we used FIEQ to explore the potential role of demographic characteristics in the evaluation of factor importance. Results showed no difference in factor importance between males and females. However, individuals with different educational levels reported significantly different evaluation scores of factor importance in three factors. This study provides a comprehensive construct of influencing factors associated with COVID-19 vaccination intention. As such, it offers important insights that could assist public health workers in promoting vaccination. Furthermore, the multifaceted nature of vaccination uptake requires attention to organizational and educational aspects, as they were crucial for the awareness and accessibility of vaccination programs (64, 65). This study would provide valuable insights into vaccination promotion strategies and offer personalized information for the development of targeted approaches.

## Data availability statement

The raw data supporting the conclusions of this article will be made available by the authors, without undue reservation.

## Ethics statement

The studies involving human participants were reviewed and approved by the Ethics Committee of the Institute of Psychology, Chinese Academy of Sciences. The patients/participants provided their written informed consent to participate in this study.

## Author contributions

YS, TZ, and SL were responsible for study design. YS and TZ was responsible for data collection. YS and SL were responsible for data analysis. YS, SL, and JX were responsible for data interpretation. YS was responsible for first draft of the manuscript JX, SL, AL, and TZ contributed to the final draft. All authors contributed to the article and approved the submitted version.

## Funding

This study was supported by the Scientific Foundation of Institute of Psychology, Chinese Academy of Sciences, No. E2CX4735YZ.

## Conflict of interest

The authors declare that the research was conducted in the absence of any commercial or financial relationships that could be construed as a potential conflict of interest.

## Publisher’s note

All claims expressed in this article are solely those of the authors and do not necessarily represent those of their affiliated organizations, or those of the publisher, the editors and the reviewers. Any product that may be evaluated in this article, or claim that may be made by its manufacturer, is not guaranteed or endorsed by the publisher.

## Footnotes

^1^ www.idiaoyan.com

## Abbreviations

AGFI: adjusted goodness of fit index
ANOVA: Analysis of variance
AVE: average variance extracted
CFA: confirmatory factor analysis
EFA: exploratory factor analysis
GFI: goodness of fit index
FIEQ: Factor Importance Evaluation Questionnaire for COVID-19 vaccine
KMO test: Kaiser–Meyer–Olkin test
RMSEA: root mean square error of approximation
SPSS: Statistical Product Service Solutions
SPSSAU: Statistical Product and Service Software Automatically
SRMR: standardized root mean square residual
