# Supplementary material for: Measuring the importance of influencing factor for COVID-19 vaccination intention in China
Source: Front Public Health. 2023 Jun 27;11:1191401. doi: 10.3389/fpubh.2023.1191401 (PMC10335563; doi:10.3389/fpubh.2023.1191401)
Supplement: Supplementary file 1 [file Table_1.docx]

Supplementary Material

Measuring the Importance of Influencing Factor for COVID-19 Vaccination Intention in China

Yue Su, Sijia Li, Jia Xue*, Ang Li, Tingshao Zhu*

*** Correspondence:**Tingshao Zhu(tszhu@psych.ac.cn) and Jia Xue(jia.xue@utoronto.ca)

# Supplementary Tables

**Appendix 1**. The first draft of FIEQ in Chinese with English translations

| **No** | **Item** | **Chinese version** |
| --- | --- | --- |
| 1 | I am very concerned about the safety of COVID-19 vaccine and whether other people have reported some side effects or adverse reactions after vaccination. | 我非常关心新冠疫苗的安全性，以及其他人接种后是否有反馈一些副作用或者不良反应。 |
| 2 | If there are reports proving that the actual effective protection rate of COVID-19 vaccine is very high, then my willingness to vaccinate will be greatly improved. | 如果有报道证明新冠疫苗实际的有效保护率很高，那么我的接种意愿将会大大提高。 |
| 3 | If vaccination takes a lot of time in queuing and traffic, I don't really want to get vaccinated. | 如果接种疫苗需要花费很多的时间在排队和交通等方面，那么我就不太想接种。 |
| 4 | I hope that news or reports can clearly state the contraindications of COVID-19 vaccine and corresponding recommendations (such as whether pregnant women can be vaccinated). | 我希望有新闻或者报道能够明确说明新冠疫苗的禁忌症和对应的建议（如孕妇是否可以接种）。 |
| 5 | I am very concerned about the specific chemical composition of COVID-19 vaccine. | 我非常关心疫苗具体的化学成分。 |
| 6 | I hope that specific explanations to certain people who have vaccination restrictions could be given when promoting COVID-19 vaccine, such as the reasons why older adults were not allowed to vaccinate before. | 我希望在推广疫苗时能够对一些有疫苗接种限制的人群给出具体的解释，比如此前不允许老人接种的原因。 |
| 7 | Compared to the official media, I prefer to believe in the opinions of some self-media figures, who have a certain number of fans and engaged in science-related content, or netizens who have been vaccinated against COVID-19. | 相较于官方媒体，我更愿意相信一些有一定粉丝量的从事科普相关内容的自媒体或者已经接种疫苗的网友对于新冠疫苗的观点。 |
| 8 | The government and official media's policy of advocating vaccinations against COVID-19 will increase my willingness of vaccination. | 政府和官方媒体提倡接种疫苗的政策会提高我的疫苗接种意愿。 |
| 9 | If it is difficult to make an appointment for the vaccination or there may be a charge for vaccination in the future, I will want to get the vaccination more eagerly now. | 如果疫苗很难预约或者之后可能收费接种，那么我现在会更急切地想要接种疫苗。 |
| 10 | If vaccine-related information or reports can indicate specific numbers such as the probability and effective rate of adverse reactions, then I will be more willing to vaccinate. | 如果疫苗相关的信息或者报道中能够标明不良反应发生的概率、有效率等具体的数字，那么我会更愿意去接种疫苗。 |
| 11 | When considering whether to vaccinate, scientifically rigorous and easy-to-understand popular science information is very important to me. | 在考虑是否要接种疫苗时，科学严谨且通俗易懂的科普信息对我而言很重要。 |
| 12 | I think getting COVID-19 vaccine shows the responsibility to others and society. | 我认为接种疫苗是对他人和社会负责的表现。 |
| 13 | As a member of an organization (such as a member of the Communist Party), I will actively respond to the organization's call for "taking the lead in vaccinating against COVID-19". | 作为某个组织的成员（比如党员等），我会积极响应组织倡议的“带头接种新冠疫苗“的号召。 |
| 14 | If the domestic epidemic outbreaks again and the risk of infection increases, then my willingness to vaccinate against COVID-19 will be greatly improved. | 假如国内疫情反弹，被感染风险增加，我的新冠疫苗接种意愿将会大大提高。 |
| 15 | I am very concerned about whether most people will choose to be vaccinated when facing COVID-19 vaccine promotion. | 我很关心大多数人在面对新冠疫苗宣传时是否会选择接种疫苗。 |
| 16 | If the government, organization or school has rigid requirements or regulations for the new crown vaccine, then I will actively respond to the vaccination. | 如果政府、工作单位或学校对于接种疫苗有硬性的要求或规定，那么我会积极响应接种疫苗。 |
| 17 | When I see professional doctors and medical workers advocating and calling for vaccinations, my willingness to vaccinate will be even stronger. | 当我看到有专业的医生、医务工作者宣传和呼吁接种疫苗时，我的接种意愿会更加强烈。 |
| 18 | I am very concerned about the opinions of individuals or institutions that are considered trustworthy on whether to vaccinate against COVID-19, such as my trusted teachers, friends, or media accounts. | 我非常关心我认为很可靠的个体或机构对于是否要接种新冠疫苗的看法，比如我所信赖的老师、朋友或者媒体账号等。 |
| 19 | I am willing to actively vaccinate against COVID-19 in response to the appeals of public figures I adore or admire. | 我愿意为了响应我所喜爱或认可的公众人物的呼吁而去积极接种疫苗。 |
| 20 | If some candid information about side effects or adverse reactions are shown while promoting the benefits of COVID-19 vaccine, I would be more willing to get the vaccine. | 如果在宣传疫苗好处的同时，我也能看到一些副作用或者不良反应的比较坦诚的信息，我会更愿意去接种疫苗 |
| 21 | If someone or some news keep advising me to vaccinate, or attack an individual who has not been vaccinated yet, these circumstances will weaken my willingness to vaccinate. | 如果一直有人或者各种消息劝我接种疫苗，或者攻击暂时没有接种疫苗的个体，这些情况会减弱我接种疫苗的意愿。 |
| 22 | If I learn that I will face the risk of infection if I do not receive the vaccine within a month, then I will be more active in getting the vaccine. | 如果我得知在一个月内不接种疫苗我将面对感染风险，那么我会更加积极地去接种疫苗。 |
| 23 | If my Health Code will become different after being vaccinated with the COVID-19 vaccine, then I will be more willing to get the vaccine. | 如果接种疫苗后，我的健康码会变得与众不同，我会更愿意去接种疫苗。 |
| 24 | I am very concerned about other benefits or conveniences other than epidemic prevention after being vaccinated against COVID-19, such as contributing to evaluation, receiving discount coupons, and making it easier to enter and exit communities or schools. | 我对于接种疫苗后，除防疫之外的其他好处或便利非常关心，如利于评优、发放优惠券、出入小区或学校更加便利等。 |
| 25 | If vaccinating against COVID-19 has other benefits to my body besides avoiding infection, I would be more willing to vaccinate. | 如果接种疫苗除了避免感染疾病之外还对身体有其他的好处，我会更愿意接种。 |
| 26 | When considering vaccination, if there are multiple options, such as one-shot, two-shot, and three-shot vaccines, I would be more willing to vaccinate. | 在考虑接种疫苗时，如果有多种选择，如一针、两针、三针的疫苗都有，我会更愿意去接种疫苗。 |
| 27 | Whether China-made COVID-19 vaccine is recognized internationally or not is very important for me to decide on vaccination. | 国际上是不是也认可我们的疫苗，这对我做判断很重要。 |
| 28 | I do hope that I can refer to the experience shared by people who have already vaccinated about the entire process of appointment, vaccination, and observation. | 我非常希望可以参考已经接种过疫苗的人分享的有关预约、接种、留观全过程的经验。 |
| 29 | When my company or group promotes the COVID-19 vaccination rate as a performance, I will be more proactive in vaccination. | 当我所在的公司或团体将疫苗接种率作为一种绩效进行宣传时，我会更加主动地去接种疫苗。 |
| 30 | If the application scope of COVID-19 vaccine and the description of adverse reactions are very explicit and clear, I will be more willing to vaccinate. | 如果新冠疫苗的适用范围和不良反应描述的非常明确和清晰，我会更愿意去接种疫苗。 |
| 31 | If I learn that my family, friends or other close people have been vaccinated against COVID-19, my willingness to be vaccinated will greatly increase. | 如果我得知自己的家人、朋友或其他身边的人接种了疫苗，我接种疫苗的意愿会大大提高。 |

**Appendix 2**. The descriptive information of 31 questions

| **Item** | **Max** | **Min** | **Mean ± SD** |
| --- | --- | --- | --- |
| 1. I am very concerned about the safety of COVID-19 vaccine and whether other people have reported some side effects or adverse reactions after vaccination. | 7 | 1 | 6.113±0.991 |
| 1. If there are reports proving that the actual effective protection rate of COVID-19 vaccine is very high, then my willingness to vaccinate will be greatly improved. | 7 | 3 | 6.399±0.8 |
| 1. If vaccination takes a lot of time in queuing and traffic, I don't really want to get vaccinated. | 7 | 1 | 4.312±1.818 |
| 1. I hope that news or reports can clearly state the contraindications of COVID-19 vaccine and corresponding recommendations (such as whether pregnant women can be vaccinated). | 7 | 1 | 6.125±0.942 |
| 1. I am very concerned about the specific chemical composition of COVID-19 vaccine. | 7 | 1 | 5.298±1.333 |
| 1. I hope that specific explanations to certain people who have vaccination restrictions could be given when promoting COVID-19 vaccine, such as the reasons why older adults were not allowed to vaccinate before. | 7 | 1 | 6.04±0.967 |
| 1. Compared to the official media, I prefer to believe in the opinions of some self-media figures, who have a certain number of fans and engaged in science-related content, or netizens who have been vaccinated against COVID-19. | 7 | 1 | 4.73±1.817 |
| 1. The government and official media's policy of advocating vaccinations against COVID-19 will increase my willingness of vaccination. | 7 | 2 | 6.116±0.953 |
| 1. If it is difficult to make an appointment for the vaccination or there may be a charge for vaccination in the future, I will want to get the vaccination more eagerly now. | 7 | 1 | 5.714±1.17 |
| 1. If vaccine-related information or reports can indicate specific numbers such as the probability and effective rate of adverse reactions, then I will be more willing to vaccinate. | 7 | 2 | 5.932±0.972 |
| 1. When considering whether to vaccinate, scientifically rigorous and easy-to-understand popular science information is very important to me. | 7 | 2 | 6.213±0.849 |
| 1. I think getting COVID-19 vaccine shows the responsibility to others and society. | 7 | 2 | 6.286±0.892 |
| 1. As a member of an organization (such as a member of the Communist Party), I will actively respond to the organization's call for "taking the lead in vaccinating against COVID-19". | 7 | 1 | 5.983±1.067 |
| 1. If the domestic epidemic outbreaks again and the risk of infection increases, then my willingness to vaccinate against COVID-19 will be greatly improved. | 7 | 1 | 6.286±0.854 |
| 1. I am very concerned about whether most people will choose to be vaccinated when facing COVID-19 vaccine promotion. | 7 | 1 | 5.704±1.179 |
| 1. If the government, organization or school has rigid requirements or regulations for the new crown vaccine, then I will actively respond to the vaccination. | 7 | 1 | 6.09±1.01 |
| 1. When I see professional doctors and medical workers advocating and calling for vaccinations, my willingness to vaccinate will be even stronger. | 7 | 3 | 6.172±0.862 |
| 1. I am very concerned about the opinions of individuals or institutions that are considered trustworthy on whether to vaccinate against COVID-19, such as my trusted teachers, friends, or media accounts. | 7 | 1 | 5.627±1.253 |
| 1. I am willing to actively vaccinate against COVID-19 in response to the appeals of public figures I adore or admire. | 7 | 1 | 5.36±1.512 |
| 1. If some candid information about side effects or adverse reactions are shown while promoting the benefits of COVID-19 vaccine, I would be more willing to get the vaccine. | 7 | 1 | 5.986±0.995 |
| 1. If someone or some news keep advising me to vaccinate, or attack an individual who has not been vaccinated yet, these circumstances will weaken my willingness to vaccinate. | 7 | 1 | 4.905±1.506 |
| 1. If I learn that I will face the risk of infection if I do not receive the vaccine within a month, then I will be more active in getting the vaccine. | 7 | 1 | 6.215±0.973 |
| 1. If my Health Code will become different after being vaccinated with the COVID-19 vaccine, then I will be more willing to get the vaccine. | 7 | 1 | 5.631±1.343 |
| 1. I am very concerned about other benefits or conveniences other than epidemic prevention after being vaccinated against COVID-19, such as contributing to evaluation, receiving discount coupons, and making it easier to enter and exit communities or schools. | 7 | 1 | 5.724±1.317 |
| 1. If vaccinating against COVID-19 has other benefits to my body besides avoiding infection, I would be more willing to vaccinate. | 7 | 1 | 6.224±0.928 |
| 1. When considering vaccination, if there are multiple options, such as one-shot, two-shot, and three-shot vaccines, I would be more willing to vaccinate. | 7 | 1 | 5.849±1.118 |
| 1. Whether China-made COVID-19 vaccine is recognized internationally or not is very important for me to decide on vaccination. | 7 | 1 | 5.475±1.399 |
| 1. I do hope that I can refer to the experience shared by people who have already vaccinated about the entire process of appointment, vaccination, and observation. | 7 | 1 | 5.983±0.988 |
| 1. When my company or group promotes the COVID-19 vaccination rate as a performance, I will be more proactive in vaccination. | 7 | 1 | 5.714±1.316 |
| 1. If the application scope of COVID-19 vaccine and the description of adverse reactions are very explicit and clear, I will be more willing to vaccinate. | 7 | 2 | 6.224±0.815 |
| 1. If I learn that my family, friends or other close people have been vaccinated against COVID-19, my willingness to be vaccinated will greatly increase. | 7 | 2 | 6.239±0.885 |

**Appendix 3.** The revised FIEQ with 20 items in Study 2

| **No** | **Item** |
| --- | --- |
| 1 | I am very concerned about the safety of COVID-19 vaccine and whether other people have reported some side effects or adverse reactions after vaccination. |
| 2 | If there are reports proving that the actual effective protection rate of COVID-19 vaccine is very high, then my willingness to vaccinate will be greatly improved. |
| 3 | I hope that news or reports can clearly state the contraindications of COVID-19 vaccine and corresponding recommendations (such as whether pregnant women can be vaccinated). |
| 4 | I hope that specific explanations to certain people who have vaccination restrictions could be given when promoting COVID-19 vaccine, such as the reasons why older adults were not allowed to vaccinate before. |
| 5 | Compared to the official media, I prefer to believe in the opinions of some self-media figures, who have a certain number of fans and engaged in science-related content, or netizens who have been vaccinated against COVID-19. |
| 6 | The government and official media's policy of advocating vaccinations against COVID-19 will increase my willingness of vaccination. |
| 7 | I think getting COVID-19 vaccine shows the responsibility to others and society. |
| 8 | If the domestic epidemic outbreaks again and the risk of infection increases, then my willingness to vaccinate against COVID-19 will be greatly improved. |
| 9 | I am very concerned about whether most people will choose to be vaccinated when facing COVID-19 vaccine promotion. |
| 10 | If the government, organization or school has rigid requirements or regulations for the new crown vaccine, then I will actively respond to the vaccination. |
| 11 | I am very concerned about the opinions of individuals or institutions that are considered trustworthy on whether to vaccinate against COVID-19, such as my trusted teachers, friends, or media accounts. |
| 12 | I am willing to actively vaccinate against COVID-19 in response to the appeals of public figures I adore or admire. |
| 13 | If some candid information about side effects or adverse reactions are shown while promoting the benefits of COVID-19 vaccine, I would be more willing to get the vaccine. |
| 14 | If my Health Code will become different after being vaccinated with the COVID-19 vaccine, then I will be more willing to get the vaccine. |
| 15 | I am very concerned about other benefits or conveniences other than epidemic prevention after being vaccinated against COVID-19, such as contributing to evaluation, receiving discount coupons, and making it easier to enter and exit communities or schools. |
| 16 | If vaccinating against COVID-19 has other benefits to my body besides avoiding infection, I would be more willing to vaccinate. |
| 17 | Whether China-made COVID-19 vaccine is recognized internationally or not is very important for me to decide on vaccination. |
| 18 | When my company or group promotes the COVID-19 vaccination rate as a performance, I will be more proactive in vaccination. |
| 19 | If the application scope of COVID-19 vaccine and the description of adverse reactions are very explicit and clear, I will be more willing to vaccinate. |
| 20 | If I learn that my family, friends or other close people have been vaccinated against COVID-19, my willingness to be vaccinated will greatly increase. |

**Appendix 4**. The mean and standard deviation of 20 items in FIEQ

| **Item** | **Max** | **Min** | **Mean ± SD** |
| --- | --- | --- | --- |
| 1. I am very concerned about the safety of COVID-19 vaccine and whether other people have reported some side effects or adverse reactions after vaccination. | 7 | 1 | 5.832±1.29 |
| 1. If there are reports proving that the actual effective protection rate of COVID-19 vaccine is very high, then my willingness to vaccinate will be greatly improved. | 7 | 2 | 6.466±0.76 |
| 1. I hope that news or reports can clearly state the contraindications of COVID-19 vaccine and corresponding recommendations (such as whether pregnant women can be vaccinated). | 7 | 1 | 6.237±0.85 |
| 1. I hope that specific explanations to certain people who have vaccination restrictions could be given when promoting COVID-19 vaccine, such as the reasons why older adults were not allowed to vaccinate before. | 7 | 1 | 6.064±0.963 |
| 1. Compared to the official media, I prefer to believe in the opinions of some self-media figures, who have a certain number of fans and engaged in science-related content, or netizens who have been vaccinated against COVID-19. | 7 | 1 | 4.447±1.879 |
| 1. The government and official media's policy of advocating vaccinations against COVID-19 will increase my willingness of vaccination. | 7 | 1 | 6.172±0.879 |
| 1. I think getting COVID-19 vaccine shows the responsibility to others and society. | 7 | 1 | 6.496±0.784 |
| 1. If the domestic epidemic outbreaks again and the risk of infection increases, then my willingness to vaccinate against COVID-19 will be greatly improved. | 7 | 1 | 6.269±1.006 |
| 1. I am very concerned about whether most people will choose to be vaccinated when facing COVID-19 vaccine promotion. | 7 | 1 | 5.461±1.231 |
| 1. If the government, organization or school has rigid requirements or regulations for the new crown vaccine, then I will actively respond to the vaccination. | 7 | 1 | 6.157±0.979 |
| 1. I am very concerned about the opinions of individuals or institutions that are considered trustworthy on whether to vaccinate against COVID-19, such as my trusted teachers, friends, or media accounts. | 7 | 1 | 5.136±1.382 |
| 1. I am willing to actively vaccinate against COVID-19 in response to the appeals of public figures I adore or admire. | 7 | 1 | 5.213±1.526 |
| 1. If some candid information about side effects or adverse reactions are shown while promoting the benefits of COVID-19 vaccine, I would be more willing to get the vaccine. | 7 | 1 | 5.809±1.121 |
| 1. If my Health Code will become different after being vaccinated with the COVID-19 vaccine, then I will be more willing to get the vaccine. | 7 | 1 | 5.594±1.313 |
| 1. I am very concerned about other benefits or conveniences other than epidemic prevention after being vaccinated against COVID-19, such as contributing to evaluation, receiving discount coupons, and making it easier to enter and exit communities or schools. | 7 | 1 | 5.67±1.29 |
| 1. If vaccinating against COVID-19 has other benefits to my body besides avoiding infection, I would be more willing to vaccinate. | 7 | 1 | 6.298±0.96 |
| 1. Whether China-made COVID-19 vaccine is recognized internationally or not is very important for me to decide on vaccination. | 7 | 1 | 5.103±1.638 |
| 1. When my company or group promotes the COVID-19 vaccination rate as a performance, I will be more proactive in vaccination. | 7 | 1 | 5.43±1.46 |
| 1. If the application scope of COVID-19 vaccine and the description of adverse reactions are very explicit and clear, I will be more willing to vaccinate. | 7 | 1 | 6.148±0.953 |
| 1. If I learn that my family, friends or other close people have been vaccinated against COVID-19, my willingness to be vaccinated will greatly increase. | 7 | 1 | 6.136±0.935 |
